# Unhealthy food consumption and asthma in children and adolescents

**DOI:** 10.1111/pai.70460

**Published:** 2026-08-14

**Authors:** Daniil Lisik, Qinyun Lin, Marika Dello Russo, Emma Goksör, Göran Wennergren, Anne‐Sophie Merritt, Inger Kull, Maike Wolters, Lauren Lissner, Sandra Ekström

**Affiliations:** ^1^ Krefting Research Centre, Institute of Medicine Sahlgrenska Academy, University of Gothenburg Gothenburg Sweden; ^2^ Department of Public Health and Clinical Medicine, the OLIN and Sunderby Research Unit Umeå University Umeå Sweden; ^3^ School of Public Health and Community Medicine, Institute of Medicine Sahlgrenska Academy, University of Gothenburg Gothenburg Sweden; ^4^ Institute of Food Sciences Consiglio Nazionale Delle Ricerche Avellino Italy; ^5^ Department of Paediatrics Sahlgrenska Academy, University of Gothenburg Gothenburg Sweden; ^6^ Centre for Occupational and Environmental Medicine Region Stockholm Stockholm Sweden; ^7^ Institute of Environmental Medicine Karolinska Institutet Stockholm Sweden; ^8^ Department of Clinical Science and Education Södersjukhuset, Karolinska Institutet Stockholm Sweden; ^9^ Sachs' Children and Youth Hospital Södersjukhuset Stockholm Sweden; ^10^ Department of Epidemiological Methods and Etiological Research Leibniz Institute for Prevention Research and Epidemiology—BIPS Bremen Germany

**Keywords:** adolescents, asthma, children, cohorts, incidence, ultra‐processed foods, unhealthy food consumption

To the Editor,

Although the prevalence of asthma in children appears to have plateaued in many European countries, including Sweden,^1^ it remains a major health challenge. Childhood asthma is associated with increased risk of various adverse outcomes, including development of COPD later in life.^2^ Thus, it is essential to elucidate actionable risk factors and thereby to inform asthma prevention strategies. Unhealthy foods, such as ultra‐processed foods (UPFs), have gained increased attention in this regard, as several reports indicate that beyond affecting metabolic and cardiovascular aspects of health, UPFs and (other) unhealthy foods may be associated with asthma.^3^, ^4^, ^5^ Most studies on this topic, however, are cross‐sectional,^6^ rendering causal inference difficult. Further, it is well‐known that the classification of processing levels in food is imperfect,^7^ with some UPFs not inherently being unhealthy and conversely some unhealthy foods not being classified as such. This is further complicated from a broader consumption pattern perspective. To expand on previous works, which have generally focused on individual foods, we assessed the longitudinal association between consumption of selected unhealthy food *groups* and asthma in children and adolescents.

We used data from three Swedish population‐based cohorts (Figure 1): (1) the Children of Western Sweden study (CWS)^8^; (2) the Child (Swedish: *Barn*), Allergy, Milieu, Stockholm, Epidemiology (BAMSE) study^9^; (3) IDEFICS (the Identification and prevention of Dietary‐ and lifestyle‐induced health Effects in Children and infantS) and I. Family (extension and follow‐up of IDEFICS) studies (IDEFICS/I. Family cohort).^10^ Through in‐person examinations/interviews and postal surveys/web questionnaires, we obtained weekly consumption frequency (irrespective of quantity per occasion of consumption) of various foods. These data were reported by the parents in CWS, by the parents/parents together with the child in BAMSE, by the parents in IDEFICS, and by the adolescents in I. Family. Then, we summed the weekly consumption frequencies of constituent food items into composite frequencies (constituting the exposure) of three predefined unhealthy food groups: sweet drinks, fast foods, and sweet/salty snacks (see Table S1 for details). As an example, for sweet drinks in CWS, a child would get the composite frequency of 1.5 if the consumption of juice was 1/week (converted numerical value 1) and the consumption of soda was <1/week (0.5). These three groups reflect three distinct patterns of unhealthy eating, covering beverages, meals, and snacks. The primary exposure was defined at a baseline assessment point in each cohort, at approximately 4.5 years (range 4–5 years) in CWS, 7–10 years (median 8.4 years) in BAMSE, and 2–9 years (median 5.9 years) in IDEFICS/I. Family. In IDEFICS/I. Family, we additionally performed sensitivity analyses using food consumption assessed at the first follow‐up (median age 7.9 years) to predict incident asthma at the second follow‐up (median age 11.2 years). The main outcome was incident parental‐reported physician diagnosis of asthma (at approximately 8 and 12 years in CWS, at [median age] 7.9 and 11.2 years in IDEFICS/I. Family, and at [median age] 13.1 and 16.6 years in BAMSE). We also assessed prevalent asthma at baseline and the previously mentioned follow‐ups.

**FIGURE 1:**
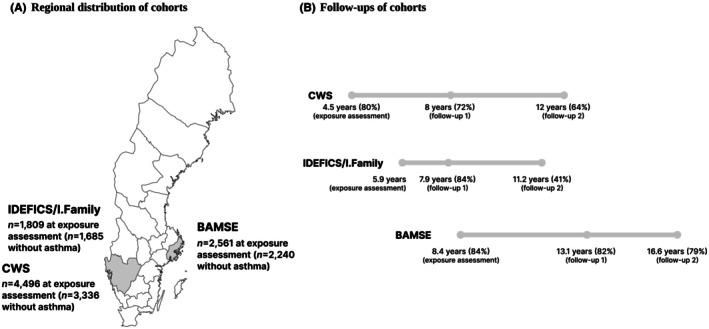
Regional distribution (A) and follow‐up periods of cohorts included in the present study (B). BAMSE: The Child (Swedish: *Barn*), Allergy, Milieu, Stockholm, Epidemiology study. CWS: The Children of Western Sweden study. IDEFICS/I. Family: The Identification and prevention of Dietary‐ and lifestyle‐induced health Effects in Children and infantS study and I. Family cohort (extension and follow‐up of IDEFICS). The catchment area of CWS and IDEFICS/I. Family was western Sweden (the Region of Västra Götaland) and for BAMSE, the Stockholm area. The percentages denote the follow‐up participation (retention) at each of the follow‐ups. Note that for CWS and BAMSE, the assessment point was also a follow‐up of said cohort.

The incidence analyses were performed using log‐binomial regression models and the prevalence analyses were performed using logistic regression models. Both analyses were run using the exposure defined in two ways: (1) as a continuous variable (i.e., the association of asthma incidence or prevalence with increasing the weekly consumption of said food group by one time); (2) as cohort‐specific tertiles (i.e., the association with the second and third tertile, respectively, using the lowest exposure category as reference). All analyses were performed on a complete‐case basis. In the incidence analyses, subjects with physician‐diagnosed asthma at baseline were excluded. All analyses were performed with and without adjustment. Covariates were selected based on extant literature and available data in each cohort, including factors such as sex, prenatal smoking exposure, birth weight, breastfeeding, and parental education (see Table S1 for details).

Baseline characteristics of the subjects in each cohort can be found in Table S2. At baseline, the number of subjects without diagnosed asthma was 3336 in CWS, 1685 in IDEFICS/I. Family, and 2240 in BAMSE. Across the two follow‐up examinations, a total of 177 (5.3%) in CWS, 44 (2.6%) in IDEFICS/I. Family, and 184 (8.2%) in BAMSE who were asthma‐free at baseline received a physician diagnosis of asthma. Figure 2 shows the results, as forest plots, of the regression analyses for asthma incidence by food group. In CWS, sweet drinks as a continuous variable were weakly associated with asthma incidence at both follow‐ups (risk ratio [RR] 1.05, 95% confidence interval [95% CI] 1.02–1.08 at age 8 years and RR 1.04, 95% CI 1.02–1.07 at age 12 years). The third tertile of sweet drinks consumption (more than six times per week, compared to one and a half times per week in the first tertile) was also significantly associated with incident asthma (RR 2.55, 95% CI 1.29–5.03 at age 8 years and RR 2.61, 95% CI 1.59–4.29 at age 12 years). For BAMSE and IDEFICS/I. Family, findings were generally nonsignificant, with mixed patterns of increased and decreased risk (Figure 2, Tables S3 and S4). However, in IDEFICS/I. Family, sweet drinks as a continuous variable were, at the first follow‐up, as in CWS, also associated with increased asthma incidence, although marginally so (RR 1.07, 95% CI 1.00–1.13). The association with prevalent asthma was mostly statistically nonsignificant for the three food groups, particularly cross‐sectionally and with the exposure in tertile form. No statistically significant associations were observed in the IDEFICS/I. Family sensitivity analyses, neither for sweet drinks or fast food, nor for sweet/salty snacks, and these findings were consistent with the overall conclusions of the primary analyses in IDEFICS/I.Family.

**FIGURE 2:**
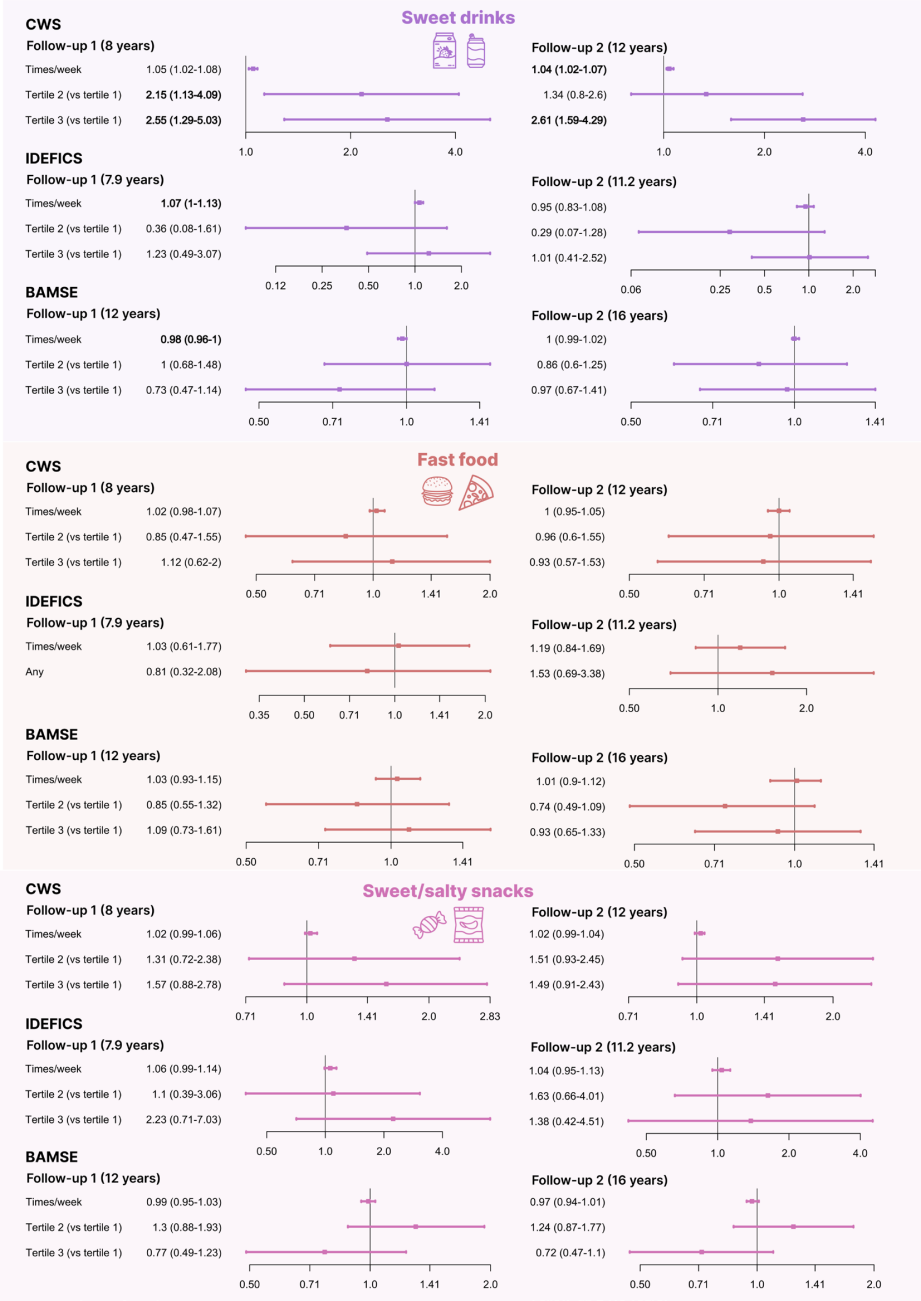
Results of regression analyses showing forest plots of incidence of asthma by unhealthy food group and cohort at 1st (left) and 2nd follow‐up (right). All results are presented as risk ratio (95% confidence interval). See Table S1 for details on exposure definitions and covariates. BAMSE, The Child (Swedish: *Barn*), Allergy, Milieu, Stockholm, Epidemiology study; CWS, The Children of Western Sweden study; IDEFICS/I. Family, The Identification and prevention of Dietary‐ and lifestyle‐induced health Effects in Children and infantS study and I. Family (extension and follow‐up of IDEFICS); RR, risk ratio.

Our findings do not support a consistent association between unhealthy food consumption overall and asthma. However, the repeated positive associations observed with sweet drink consumption across two cohorts warrant further investigation. While some previous studies suggest an association between unhealthy foods and asthma, they were mostly cross‐sectional,^6^ in which causality is difficult to establish. Our study, in contrast, makes use of relatively long‐term longitudinal data with two follow‐ups. The significant associations were seen for sweet drinks. These findings coincide with similar cross‐sectional studies, in which associations have been reported to be among the strongest for such beverages.^11^ In cross‐sectional studies, the results generally point in the same direction as our results and are overall stronger, which may, collectively, strengthen the hypothesis that the association is there. The weaker associations observed in our data may also be due, in part, to exposure misclassification, as unhealthy food consumption patterns assessed at a single time point may have changed over time, although the longitudinal design ensures the correct temporal ordering between exposure and outcome. Importantly, even the strongest associations in our data, for sweet drinks as exposure, were mostly limited to CWS, and to some extent IDEFICS/I. Family. This may reflect genuine cohort‐specific differences, for example, due to unmeasured confounders or differences in dietary assessment, but it may also relate to differences in the age at exposure, as CWS and IDEFICS baseline age were lower than BAMSE. In regard to sweet/salty snacks and fast foods, the nonsignificant results may be due to large heterogeneity in the constituent food item categories, in contrast to sweet drinks, which were mostly defined as juice and soda. Food consumption is complex and multifaceted. Associations may differ by specific food items and the overall eating patterns and lifestyle. Beyond inter‐cohort differences (including notable variation in assortment and numbers of constituent food items in each category as well as tertile thresholds), the examined foods could potentially constitute components in varied meals with high nutritional value accompanied by healthy living conditions, physical activity, and other lifestyle and asthma risk factors. These factors could not be fully harmonized and accounted for in this study, and therefore potentially introduced unmeasured confounding. Likewise, residual confounding is possible. It is also likely that parents do not have a comprehensive picture of what the children eat while with friends and at preschool/school.^12^ The utilized questionnaires do not comprehensively capture eating habits, and some degree of reporting bias is to be expected. Further, the timing of the exposure assessment varied between the cohorts. Given that most significant findings were seen in CWS, it is possible that the effect that exists relates to food consumption at an early age, but the difference in overlap of exposure assessment time hampered this assessment. A further limitation in the analyzed exposures is that there were limited data to explore the effects of *longitudinal* consumption patterns. Similarly, the available data did not allow for example, spirometry‐based definition of asthma.

Further studies using standardized dietary assessment across cohorts and repeated measures of food consumption are needed to improve classification of food groups and elucidate whether specific foods or combinations thereof, as well as persistent dietary patterns over time, are associated with asthma. Beyond this, more research is needed to discern whether indeed the timing of unhealthy food consumption plays a role in this context and whether repeated or persistent exposure during childhood confers greater risk than isolated measures.

## AUTHOR CONTRIBUTIONS

**Qinyun Lin:** Conceptualization; methodology; validation; writing – review and editing; investigation; formal analysis; software. **Sandra Ekström:** Formal analysis; writing – review and editing; validation; methodology; conceptualization; investigation. **Anne‐Sophie Merritt:** Writing – review and editing; validation; supervision. **Marika Dello Russo:** Validation; writing – review and editing; supervision. **Emma Goksör:** Conceptualization; validation; writing – review and editing; supervision; data curation. **Göran Wennergren:** Data curation; supervision; validation; writing – review and editing; conceptualization. **Lauren Lissner:** Data curation; project administration; supervision; visualization; conceptualization; investigation; validation; methodology; writing – review and editing. **Inger Kull:** Writing – review and editing; validation; supervision; data curation. **Maike Wolters:** Validation; writing – review and editing; supervision. **Daniil Lisik:** Conceptualization; investigation; writing – original draft; methodology; validation; visualization; writing – review and editing; software; formal analysis; project administration.

## FUNDING INFORMATION

The authors have nothing to report.

## CONFLICT OF INTEREST STATEMENT

The authors declare no conflicts of interest.

## ETHICS STATEMENT

The study was approved by the Regional Ethics Committee of Gothenburg (Ö 524‐00 and 846‐14) for CWS, the Regional Ethics Review Board at Karolinska Institutet in Stockholm (2016/1380‐31/2) for BAMSE, and the Regional Ethical Review Board in Gothenburg (176‐18) for IDEFICS/I. Family. Informed written consent was obtained from all parents/participants.

## Supporting information


**Table S1.** Methodological approaches across the included cohorts.
**Table S2:** Baseline characteristics by cohort.
**Table S3:** Results of regression analyses showing the association of unhealthy food group consumption with the incidence of physician‐diagnosed asthma.
**Table S4:** Results of regression analyses showing the association of unhealthy food group consumption with the prevalence of physician‐diagnosed asthma.

## Data Availability

Due to local regulations and contractual agreement with study participants, the underlying data are not publicly available for CWS and BAMSE. Access to and use of IDEFICS/I. Family cohort data and biosamples can be obtained via an electronic application portal at https://www.bips‐institut.de/en/research/research‐data‐portal.
