# Supplementary material for: Unhealthy food consumption and asthma in children and adolescents
Source: Pediatr Allergy Immunol. 2026 Aug 14;37(8):e70460. doi: 10.1111/pai.70460 (PMC13474128; doi:10.1111/pai.70460)
Supplement: Supplementary file 1 [file PAI-37-e70460-s001.docx]

**Table S1. Methodological approaches across the included cohorts**

| **Cohort →**  **Item ↓** | **CWS** | **IDEFICS/I.Family** | **BAMSE** |
| --- | --- | --- | --- |
| **Included foods in each food group** | | | |
| **Sweet drinks** | - Juice - Soda | Sweetened drinks including sports drinks, bottled or canned tea, syrup-based drinks and similar (e.g. <local examples> etc.)  Fruit juices (orange juice, apple juice, <local examples> etc.) | - Soft drinks - Lemonade - Juice - Chocolate milk (O’boy) |
| **Fast food** | - Pizza - Hamburger - Fish sticks - Sausage/processed meat - French fries | Hamburger, hot dog, kebab, wrap, falafel <local examples> | - Pizza - French fries - Sausage - Fish sticks |
| **Sweet/salty snacks** | - Cookies, cake, pastries - Chips, popcorn, salted nuts - Ice cream - Sweets/candy | Snacks like crisps, corn crisps, popcorn etc (e.g. <local examples> etc.)  Snacks like savoury pastries and fritters (e.g. cheese pie, sausage pie, pancakes, pizza as snack, <local examples> etc.)  Snacks like chocolate, candy bars (mars, lions, kit Kat, <local examples> etc.)  Snacks like candies, loose candies, marshmallow (e.g. <local examples> etc.)  Snacks like biscuits, packaged cakes, or pastries and puddings (e.g. <local examples> etc.)  Ice cream, milk or fruit based bars (e.g. <local examples> etc.) | - Buns - Danish pastries - Biscuits/wafers/rusks - Cookies - Cakes/pastries - Chocolate - Candy - Ice-cream - Crisps - Popcorn |
| **Consumption frequency definitions** | | | |
| **Weekly consumption frequency categories as per questionnaires (numbers in parentheses denote the numerical representation used in the analyses)** | - <1/week (0.5) - 1/week (1) - 2-6/week (4) - 1-2/day (10.5) - ≥3/day (21)   Defined with the question: *“How often has your child eaten the following food items, on average, in the past month? Mark the alternative that you consider to be the most accurate”* (similar wording was used in IDEFICS/I.Family and BAMSE) | - Never/less than once a week (0)  - 1-3/week (2)  - 4-6/week (5)  - 1/day (7)  - 2/day (14)  - 3/day (21)  - 4/day (28) | - Never (0) - Less than 1 /month (0.5/30) - 1-3 times/month (0.5) - 1/week (1) - 2/week (2) - 3-4/week (3.5) - 5-6/week (5.5) - 1/day (7) - 2/day (14) - 3/day (21) |
| **Tertile thresholds, sweet drinks** | ≤1.5, >1.5 and ≤ 5, >5 | ≤4, >4 and ≤8, >8 | <7, 7-12.5, >12.5 |
| **Tertile thresholds, fastfood** | ≤ 3, >3 and ≤ 6, >6 | Fast food consumption was highly zero-inflated; therefore, it was categorized as any vs none. | <2.41, 2.47-3.4, >3.5 |
| **Tertile thresholds, sweet/salty snacks** | ≤ 3.5, >3.5 and ≤ 6.5, >6.5 | ≤2, >2 and ≤5, >5 | <7.12, 7.12-10.95, >10.96 |
| **Covariates** | | | |
| **Covariates included** | - Sex - Prenatal maternal smoking - Maternal education - Birth weight - Breastfeeding (≥ 3 months) - Neonatal antibiotics - Parental asthma - Introduction to fish before age 9 months | - Sex  - Age at baseline  - Parental education  - Birth weight  - Smoking (cigarettes) during pregnancy  - Breastfeeding (<= 3 months, 4 months, 4-8 months)  - Follow up time  - BMI z-score at baseline? | - Sex - Smoking during pregnancy - Parental education - Birth weight - Breastfeeding (≥4 months) - Follow-up time to asthma - Child’s age at baseline - BMI categories (as per the International Obesity Task Force; IOTF) at baseline |
| **Ages** | | | |
| **Age at baseline (timepoint during which exposure data were collected)** | ~4.5 years (range 4-5 years) | Median [Q1, Q3]  5.9 [3.9, 7.5] | Median [Q1, Q3]  8.4 [8.0, 8.7] |
| **Age at follow-up 1** | ~8 years | Median [Q1, Q3]  7.9 [6, 9.4] | Median [Q1, Q3]  13.1 [12.3, 13.7] |
| **Age at follow-up 2** | ~12 years | Median [Q1, Q3]  11.2 [9.5, 13] | Median [Q1, Q3]  16.6 [16.2, 16.8] |
| **Statistical analysis details** | | | |
| **Handling of missingness** | Complete-case analysis | Complete-case analysis | Complete-case analysis |
| **Regression model used for prevalence analysis** | Binary logistic regression (OR) | Binary logistic regression (OR) | Binary logistic regression (OR) |
| **Regression model used for longitudinal, incidence analysis** | Log-binomial regression with EM algorithm (RR) | Generalized linear model with binomial family and log link function (RR) | Generalized linear model with binomial family and log link function (RR) |

**Abbreviations.** BAMSE: the Child (Swedish: Barn), Allergy, Milieu, Stockholm, Epidemiological study. BMI: body mass index. CWS: the Children of Western Sweden study. IDEFICS/I.Family: the Identification and prevention of Dietary- and lifestyle-induced health Effects in Children and infantS study and I.Family (extension and follow-up of IDEFICS). OR: odds ratio. Q1: first quartile. Q3: third quartile. RR: risk ratio. **Notes.** Because the IDEFICS/I.Family cohort has a very small number of *incident* asthma cases, we fitted parsimonious models with one exposure at a time, adjusting only for sex, parental education, and prenatal smoking to maintain a reasonable events-per-variable ratio. Additional baseline variables are reported in the characteristics’ tables (Table S1 and Table S2) and were explored in sensitivity analyses (results not shown but remained consistent) but were not included in the primary models to avoid overfitting.

**Table S2. Baseline characteristics by cohort**

| **Cohort →**  **Variable ↓** | **CWS** | **IDEFICS/I.Family** | **BAMSE** |
| --- | --- | --- | --- |
| **Sex (female; n (%))** | 1,999 (48%) | 882 (48.76%) | 1,257 (49%) |
| **Age (at baseline; years)** | ~4.5 years (range 4-5 years) | Median [Q1, Q3]: 5.9 [3.9, 7.5]) | Median [Q1, Q3]: 8.4 [8.0, 8.7] |
| **Prenatal maternal smoking (n (%))** | 368 (8.3%) | 305 (17%: Never)  1,504 (83%: Rarely, at maximum once a month, several occasions a week; Daily)  This definition included cigarette smoking and the use of other tobacco products. | 310 (12%) |
| **Parental education (n (%))** | 148 (3.3%; primary)  1,989 (45%; secondary)  2,306 (52%; tertiary) | 551 (31%: low or medium)  1,223 (69%; high) | 54 (2.1%; primary)  1,128 (44%; secondary)  1,377 (54%; tertiary) |
| **Birth weight (g; mean ± SD)** | 3,575 ± 572 | 3,515.73 ± 591 | 3,533 ± 559 |
| **Breastfeeding (n (%))** | 3,162 (86%; for ≥3 months) | 611 (36%: ≤ 3 months)  657 (38%, 4 months)  448 (26%, 4-8 months) | 2,048 (81% for ≥4 months) |
| **Neonatal antibiotics (n (%))** | 197 (4.5%) | N/A | 1,076 (43%; up to age 1 year) |
| **Parental asthma (n (%))** | 741 (17%) | N/A (we only asked for ever been diagnosed lung diseases in parents (asthma, COPD) in I.Family) | 510 (20%) |
| **Early introduciton to fish (n (%))** | 3,195 (83%, before age 9 months) | N/A | 1,376 (55%; before age 9 months) |
| **Baseline BMI (kg/m^2^)** | N/A | Median [Q1, Q3]: 15.7 [14.8, 16.7] | Mean ± SD: 17.2 ± 2.2 |

**Clarifications.** Categorical variables are shown as number of subjects (% of subjects), while continuous variables are shown as mean ± standard deviation or median [Q1, Q3], where specifics of such data are available (otherwise, an approximation is given). **Abbreviations.** BAMSE: the Child (Swedish: Barn), Allergy, Milieu, Stockholm, Epidemiological study. BMI: body mass index. CWS: the Children of Western Sweden study. IDEFICS/I.Family: the Identification and prevention of Dietary- and lifestyle-induced health Effects in Children and infantS study and I.Family (extension and follow-up of IDEFICS). Q1: first quartile. Q3: third quartile. SD: standard deviation.

**Table S3. Results of regression analyses showing the association of unhealthy food group consumption with the (incidence of physician-diagnosed asthma**

| **CWS** | | | | |
| --- | --- | --- | --- | --- |
| **Weekly fast food group consumption, at age ~4.5 years, as a continuous variable** | | | | |
|  | **Incident asthma by age…** | | | |
|  | **~8 years (94 new cases)** | | **~12 years (129 new cases)** | |
|  | Crude RR (95%CI) | Adjusted RR (95%CI) | Crude RR (95%CI) | Adjusted RR (95%CI) |
| Sweet drinks (times/w) | **1.05 (1.02-1.08)** | **1.05 (1.02-1.08)** | **1.03 (1.01-1.06)** | **1.04 (1.02-1.07)** |
| Fast food (times/w) | 1.02 (0.99-1.05) | 1.02 (0.98-1.07) | 0.99 (0.95-1.04) | 1 (0.95-1.05) |
| Sweet/salty snacks (times/w) | 1.01 (0.98-1.04) | 1.02 (0.99-1.06) | 1.01 (0.99-1.04) | 1.02 (0.99-1.04) |
| Analytical sample size | 2,616-2,622 | 1,991-1,996 | 2,465-2,473 | 1,909-1,914 |
| **Food group consumption at ~4.5 years, as tertials, using tertile 1 as reference group** | | | | |
|  | **Incident asthma by age…** | | | |
|  | **~8 years (94 new cases)** | | **~12 years (129 new cases)** | |
|  | Crude RR (95%CI) | Adjusted RR (95%CI) | Crude RR (95%CI) | Adjusted RR (95%CI) |
| Sweet drinks | | | | |
| Tertile 2 (>1.5 and ≤5 times/w) | 1.52 (0.93-2.49) | **2.15 (1.13-4.09)** | 1.15 (0.76-1.74) | 1.34 (0.8-2.26) |
| Tertile 3 (>5 times/w) | **1.99 (1.18-3.36)** | **2.55 (1.29-5.03)** | **1.78 (1.18-2.7)** | **2.61 (1.59-4.29)** |
| Fast food | | | | |
| Tertile 2 (>3 and ≤6 times/w) | 0.88 (0.54-1.44) | 0.85 (0.47-1.55) | 0.91 (0.61-1.36) | 0.96 (0.6-1.55) |
| Tertile 3 (>6 times/w) | 1.17 (0.73-1.89) | 1.12 (0.62-2) | 0.99 (0.65-1.49) | 0.93 (0.57-1.53) |
| Sweet/salty snacks | | | | |
| Tertile 2 (>3.5 and ≤6.5 times/w) | 1.03 (0.63-1.67) | 1.31 (0.72-2.38) | 1.28 (0.85-1.92) | 1.51 (0.93-2.45) |
| Tertile 3 (>6.5 times/w) | 1.08 (0.67-1.73) | 1.57 (0.88-2.78) | 1.26 (0.84-1.89) | 1.49 (0.91-2.43) |
| Analytical sample size | 2,616-2,622 | 1,991-1,996 | 2,465-2,473 | 1,909-1,914 |
| **BAMSE** | | | | |
| **Weekly fast food group consumption, at age 8.4 (median) years, as a continuous variable** | | | | |
|  | **Incident asthma by age…** | | | |
|  | **13.1 (median) years (132 new cases)** | | **16.6 (median) years (184 new cases)** | |
|  | Crude RR (95%CI) | Adjusted RR (95%CI) | Crude RR (95%CI) | Adjusted RR (95%CI) |
| Sweet drinks (times/w) | 0.98 (0.96-1.01) | 0.98 (0.96-1.00) | 1.01 (0.99-1.02) | 1.00 (0.99-1.02) |
| Fast food (times/w) | 1.03 (0.92-1.14) | 1.03 (0.93-1.15) | 1.05 (0.96-1.14) | 1.01 (0.90-1.12) |
| Sweet/salty snacks (times/w) | 0.98 (0.95-1.02) | 0.99 (0.95-1.03) | 0.98 (0.95-1.01) | 0.97 (0.94-1.01) |
| Analytical sample size | 2,016-2,062 | 1,969-2,015 | 1,952-2,006 | 1,690-1,736 |
| **Food group consumption at 8.4 (median) years, as tertials, using tertile 1 as reference group** | | | | |
|  | **Incident asthma by age…** | | | |
|  | **13.1 (median) years (132 new cases)** | | **16.6 (median) years (184 new cases)** | |
|  | Crude RR (95%CI) | Adjusted RR (95%CI) | Crude RR (95%CI) | Adjusted RR (95%CI) |
| Sweet drinks | | | | |
| Tertile 2 (≥7 and ≤12.5 times/w) | 1.05 (0.71-1.55) | 1.00 (0.68-1.48) | 0.96 (0.69-1.35) | 0.91 (0.65-1.29) |
| Tertile 3 (>12.5 times/w) | 0.75 (0.49-1.17) | 0.73 (0.47-1.14) | 0.97 (0.69-1.37) | 0.99 (0.70-1.40) |
| Fast food | | | | |
| Tertile 2 (≥2.47 and ≤3.4 times/w) | 0.81 (0.52-1.25) | 0.85 (0.55-1.32) | 0.77 (0.53-1.12) | 0.83 (0.57-1.20) |
| Tertile 3 (≥3.5 times/w) | 1.04 (0.71-1.52) | 1.09 (0.73-1.61) | 1.08 (0.78-1.47) | 1.08 (0.78-1.50) |
| Sweet/salty snacks | | | | |
| Tertile 2 (≥7.12 and ≤10.95 times/w) | 1.26 (0.85-1.86) | 1.30 (0.88-1.93) | 1.18 (0.85-1.65) | 1.22 (0.87-1.71) |
| Tertile 3 (≥10.96 times/w) | 0.72 (0.46-1.15) | 0.77 (0.49-1.23) | 0.81 (0.56-1.17) | 0.79 (0.54-1.16) |
| Analytical subsample | 2,016-2,062 | 1,969-2,015 | 1,952-2,006 | 1,690-1,736 |
| **IDEFICS/I.Family** | | | | |
| **Food group consumption at 5.9 (median) years, as a continuous variable** | | | | |
|  | **Incident asthma by age…** | | | |
|  | **7.9 (median) years (25 new cases)** | | **11.2 (median) years (24 new cases)** | |
|  | Crude RR (95%CI) | Adjusted RR (95%CI) | Crude RR (95%CI) | Adjusted RR (95%CI) |
| Sweet drinks (times/w) | **1.07 (1.02, 1.13)** | **1.07 (1.00, 1.13)** | 0.96 (0.86, 1.08) | 0.95 (0.83, 1.08) |
| Fast food (times/w) | 1.02 (0.61, 1.71) | 1.03 (0.61, 1.77) | 1.18 (0.83, 1.68) | 1.19 (0.84, 1.69) |
| Sweet/salty snacks (times/w) | 1.06 (0.99, 1.14) | 1.06 (0.99, 1.14) | 1.05 (0.96, 1.15) | 1.04 (0.95, 1.13) |
| Analytical sample size | 1,353–1,361 | 1,352–1,361 | 686–692 | 686–692 |
| **Food group consumption at 5.9 (median) years, as categories, using the lowest category as reference** | | | | |
|  | **Incident asthma by age…** | | | |
|  | **7.9 (median) years (25 new cases)** | | **11.2 (median) years (24 new cases)** | |
|  | Crude RR (95% CI) | Adjusted RR (95% CI) | Crude RR (95%CI) | Adjusted RR (95%CI) |
| Sweet drinks | | | | |
| Tertile 2 (>2 and ≤5 times/w) | 0.36 (0.08, 1.59) | 0.36 (0.08, 1.61) | 0.29 (0.07, 1.31) | 0.29 (0.07, 1.28) |
| Tertile 3 (>5 times/w) | 1.26 (0.54, 2.97) | 1.23 (0.49, 3.07) | 1.14 (0.50, 2.62) | 1.01 (0.41, 2.52) |
| Fast food | | | | |
| Any (>0 times/w) | 0.79 (0.31, 2.00) | 0.81 (0.32, 2.08) | 1.50 (0.67, 3.37) | 1.53 (0.69, 3.38) |
| Sweet/salty snacks | | | | |
| Tertile 2 (>5 and ≤8 times/w) | 0.98 (0.36, 2.68) | 1.10 (0.39, 3.06) | 1.66 (0.66, 4.16) | 1.63 (0.66, 4.01) |
| Tertile 3 (>8 times/w) | 1.95 (0.72, 5.33) | 2.23 (0.71, 7.03) | 1.42 (0.46, 4.41) | 1.38 (0.42, 4.51) |
| Analytical sample size | 1,353–1,361 | 1,352–1,361 | 686–692 | 686–692 |

**Abbreviations.** BAMSE: the Child (Swedish: Barn), Allergy, Milieu, Stockholm, Epidemiological study. CWS: the Children of Western Sweden study. IDEFICS/I.Family: the Identification and prevention of Dietary- and lifestyle-induced health Effects in Children and infantS study and I.Family (extension and follow-up of IDEFICS). RR: risk ratio. w: week: 95%CI: 95% confidence interval. **Notes.** To harmonize analyses across cohorts, the primary exposure was defined at the baseline examination in each cohort. In IDEFICS/I.Family, we additionally assessed alternative exposure definitions using later examination data in sensitivity analyses; results were materially unchanged.

**Table S4. Results of regression analyses showing the association of unhealthy food group consumption with the prevalence of physician-diagnosed asthma**

| **CWS** | | | | | | |
| --- | --- | --- | --- | --- | --- | --- |
| **Weekly fast food group consumption, at age ~4.5 years, as a continuous variable** | | | | | | |
|  | **Prevalence of asthma at age…** | | | | | |
|  | **~4.5 years (284 cases)** | | **~8 years (269 cases)** | | **~12 years (263 cases)** | |
|  | Crude OR (95%CI) | Adjusted OR (95%CI) | Crude OR (95%CI) | Adjusted OR (95%CI) | Crude OR (95%CI) | Adjusted OR (95%CI) |
| Sweet drinks (times/w) | 1 (0.97-1.02) | 0.99 (0.96-1.02) | **1.02 (1-1.05)** | **1.02 (1-1.05)** | **1.02 (1-1.04)** | **1.03 (1-1.05)** |
| Fast food (times/w) | **1.02 (1-1.04)** | **1.03 (1-1.05)** | **1.03 (1-1.05)** | **1.03 (1-1.06)** | 1.02 (0.99-1.04) | 1.01 (0.98-1.05) |
| Sweet/salty snacks (times/w) | 0.99 (0.97-1.01) | 1.01 (0.98-1.03) | **0.98 (0.96-1)** | 1 (0.97-1.02) | 1 (0.98-1.02) | 1 (0.98-1.03) |
| Analytical sample size | 3,601-3,609 | 2,671-2,677 | 3,508-3,521 | 2,644-2,654 | 3,315-3,328 | 2,534-2,543 |
| **Food group consumption at ~4.5 years, as tertials, using tertile 1 as reference group** | | | | | | |
|  | **Prevalence of asthma at age…** | | | | | |
|  | **~4.5 years (284 cases)** | | **~8 years (269 cases)** | | **~12 years (263 cases)** | |
|  | Crude OR (95%CI) | Adjusted OR (95%CI) | Crude OR (95%CI) | Adjusted OR (95%CI) | Crude OR (95%CI) | Adjusted OR (95%CI) |
| Sweet drinks | | | | | | |
| Tertile 2 (>1.5 and ≤5 times/w) | 1 (0.76-1.32) | 0.94 (0.68-1.32) | 1.21 (0.9-1.61) | 1.29 (0.91-1.84) | 1.11 (0.83-1.49) | 1.03 (0.72-1.49) |
| Tertile 3 (>5 times/w) | 1.01 (0.73-1.38) | 0.98 (0.67-1.43) | 1.37 (0.99-1.9) | 1.32 (0.88-1.96) | 1.32 (0.96-1.83) | **1.5 (1.01-2.2)** |
| Fast food | | | | | | |
| Tertile 2 (>3 and ≤6 times/w) | 1.07 (0.79-1.44) | 0.86 (0.6-1.24) | 1.01 (0.75-1.38) | 0.85 (0.58-1.23) | 1.01 (0.75-1.37) | 0.85 (0.59-1.25) |
| Tertile 3 (>6 times/w) | 1.26 (0.93-1.7) | 1.16 (0.81-1.65) | 1.25 (0.92-1.69) | 1.09 (0.75-1.56) | 1.15 (0.84-1.56) | 0.96 (0.66-1.4) |
| Sweet/salty snacks | | | | | | |
| Tertile 2 (>3.5 and ≤6.5 times/w) | 0.8 (0.59-1.07) | 0.83 (0.57-1.2) | 0.91 (0.67-1.22) | 1.03 (0.71-1.48) | 1.03 (0.75-1.39) | 1.19 (0.82-1.73) |
| Tertile 3 (>6.5 times/w) | 0.82 (0.61-1.09) | 1.12 (0.79-1.57) | 0.77 (0.57-1.04) | 1.03 (0.72-1.48) | 0.95 (0.7-1.28) | 1.08 (0.74-1.57) |
| Analytical sample size | 3,601-3,609 | 2,671-2,677 | 3,508-3,521 | 2,644-2,654 | 3,315-3,328 | 2,534-2,543 |
| **BAMSE** | | | | | | |
| **Weekly fast food group consumption, at age 8.4 (median) years, as a continuous variable** | | | | | | |
|  | **Prevalence of asthma at age…** | | | | | |
|  | **8.4 (median) years (321 cases)** | | **13.1 (median) years (364 cases)** | | **16.6 (median) years (419 cases)** | |
|  | Crude OR (95%CI) | Adjusted OR (95%CI) | Crude OR (95%CI) | Adjusted OR (95%CI) | Crude OR (95%CI) | Adjusted OR (95%CI) |
| Sweet drinks (times/w) | 1.00 (0.98-1.01) | 0.99 (0.98-1.01) | 0.99 (0.97-1.00) | **0.98 (0.97-0.998)** | 1.00 (0.98-1.01) | 0.99 (0.98-1.01) |
| Fast food (times/w) | **1.09 (1.01-1.17)** | 1.06 (0.98-1.14) | 1.06 (0.99-1.14) | 1.04 (0.97-1.12) | **1.07 (1.00-1.15)** | 1.05 (0.97-1.14) |
| Sweet/salty snacks (times/w) | 1.00 (0.98-1.02) | 1.00 (0.98-1.03) | 0.99 (0.96-1.01) | 0.99 (0.97-1.01) | 0.98 (0.96-1.00) | 0.98 (0.96-1.01) |
| Analytical sample size | 2,408-2,467 | 2,352-2,409 | 2,340-2,397 | 2,280-2,335 | 2,264-2,325 | 1,946-1,997 |
| **Food group consumption at 8.4 (median) years, as tertials, using tertile 1 as reference group** | | | | | | |
|  | **Prevalence of asthma at age…** | | | | | |
|  | **8.4 (median) years (321 cases)** | | **13.1 (median) years (364 cases)** | | **16.6 (median) years (419 cases)** | |
|  | Crude OR (95%CI) | Adjusted OR (95%CI) | Crude OR (95%CI) | Adjusted OR (95%CI) | Crude OR (95%CI) | Adjusted OR (95%CI) |
| Sweet drinks | | | | | | |
| Tertile 2 (≥7 and ≤12.5 times/w) | 0.98 (0.73-1.31) | 0.98 (0.72-1.32) | 1.00 (0.76-1.31) | 0.98 (0.75-1.30) | 0.89 (0.69-1.15) | 0.86 (0.65-1.15) |
| Tertile 3 (>12.5 times/w) | 1.08 (0.73-1.31) | 1.00 (0.74-1.34) | 0.92 (0.69-1.21) | 0.82 (0.61-1.09) | 0.94 (0.72-1.22) | 0.93 (0.70-1.24) |
| Fast food | | | | | | |
| Tertile 2 (≥2.47 and ≤3.4 times/w) | 1.21 (0.89-1.65) | 1.18 (0.86-1.61) | 0.91 (0.68-1.22) | 0.90 (0.66-1.21) | 0.92 (0.70-1.21) | 0.95 (0.70-1.28) |
| Tertile 3 (≥3.5 times/w) | **1.42 (1.07-1.88)** | 1.31 (0.98-1.75) | 1.18 (0.91-1.53) | 1.14 (0.87-1.50) | 1.22 (0.95-1.56) | 1.16 (0.88-1.52) |
| Sweet/salty snacks | | | | | | |
| Tertile 2 (≥7.12 and ≤10.95 times/w) | 1.09 (0.82-1.45) | 1.14 (0.85-1.53) | 1.10 (0.84-1.45) | 1.15 (0.87-1.51) | 1.11 (0.86-1.44) | 1.13 (0.85-1.49) |
| Tertile 3 (≥10.96 times/w) | 0.93 (0.69-1.25) | 0.96 (0.70-1.30) | 0.77 (0.57-1.02) | 0.80 (0.60-1.08) | **0.75 (0.57-0.98)** | **0.73 (0.54-0.99)** |
| Analytical sample size | 2,408-2,467 | 2,352-2,409 | 2,340-2,397 | 2,280-2,335 | 2,264-2,325 | 1,946-1,997 |
| **IDEFICS/I.Family** | | | | | | |
| **Food group consumption at 5.9 (median) years, as a continuous variable.** | | | | | | |
|  | **Prevalence of asthma at age…** | | | | | |
|  | **5.9 (median) years (123 cases)** | | **7.9 (median) years (97 cases)** | | **11.2 (median) years (49 cases)** | |
|  | Crude OR (95%CI) | Adjusted OR (95%CI) | Crude OR (95%CI) | Adjusted OR (95%CI) | Crude OR (95%CI) | Adjusted OR (95%CI) |
| Sweet drinks (times/w) | 1.01(0.97, 1.05) | 1.00 (0.96, 1.05) | 1.02(0.98, 1.07) | 1.02(0.97, 1.07) | 1.01(0.95, 1.08) | 1.00(0.93, 1.07) |
| Fast food (times/w) | 0.95(0.79, 1.16) | 0.95(0.78, 1.15) | 0.92(0.74, 1.16) | 0.91(0.73, 1.14) | 0.91(0.65, 1.26) | 0.92(0.66, 1.28) |
| Sweet/salty snacks (times/w) | 1.00(0.95, 1.05) | 0.99 (0.94, 1.04) | 1.00(0.94, 1.06) | 0.99(0.94, 1.05) | 1.01(0.93, 1.10) | 1.01(0.92, 1.09) |
| Analytical sample size | 1,733–1,745 | 1,730–1,742 | 1,454–1,464 | 1,370–1,462 | 736–743 | 736–743 |
| **Food group consumption at 5.9 (median) years, as categories, using the lowest category as reference.** | | | | | | |
|  | **Prevalence of asthma at age…** | | | | | |
|  | **5.9 (median) years (123 cases)** | | **7.9 (median) years (97 cases)** | | **11.2 (median) years (49 cases)** | |
|  | Crude OR (95%CI) | Adjusted OR (95%CI) | Crude OR (95%CI) | Adjusted OR (95%CI) | Crude OR (95%CI) | Adjusted OR (95%CI) |
| Sweet drinks | | | | | | |
| Tertile 2 (>2 and ≤5 times/w) | 0.86(0.53, 1.41) | 0.82(0.50, 1.35) | 0.57(0.31, 1.04) | 0.56(0.30, 1.02) | **0.22(0.07, 0.74)** | **0.22(0.06, 0.73)** |
| Tertile 3 (>5 times/w) | 1.06(0.69, 1.62) | 0.95(0.62, 1.46) | 0.94(0.58, 1.50) | 0.86(0.54, 1.40) | 1.28(0.69, 2.35) | 1.11(0.60, 2.08) |
| Fast food | | | | | | |
| Any (>0 times/w) | 0.94(0.63, 1.41) | 0.92(0.61, 1.39) | 0.81(0.50, 1.29) | 0.78(0.49, 1.26) | 0.84(0.43, 1.64) | 0.86(0.44, 1.71) |
| Sweet/salty snacks | | | | | | |
| Tertile 2 (>5 and ≤8 times/w) | 0.98(0.65, 1.48) | 0.94(0.62, 1.42) | 0.93(0.58, 1.48) | 0.90(0.56, 1.44) | 0.97(0.50, 1.87) | 0.94(0.48, 1.83) |
| Tertile 3 (>8 times/w) | 0.95(0.57, 1.58) | 0.87(0.52, 1.46) | 0.96(0.54, 1.68) | 0.90(0.51, 1.59) | 1.18(0.55, 2.55) | 1.12(0.51, 2.46) |
| Analytical sample size | 1,733–1,745 | 1,730–1,742 | 1,454–1,464 | 1,370–1,462 | 736–743 | 736–743 |

**Abbreviations.** BAMSE: the Child (Swedish: Barn), Allergy, Milieu, Stockholm, Epidemiological study. CWS: the Children of Western Sweden study. IDEFICS/I.Family: the Identification and prevention of Dietary- and lifestyle-induced health Effects in Children and infantS study and I.Family (extension and follow-up of IDEFICS). OR: odds ratio. w: week. 95%CI: 95% confidence interval. **Notes.** To harmonize analyses across cohorts, the primary exposure was defined at the baseline examination in each cohort. In IDEFICS/IFamily, we additionally assessed alternative exposure definitions using later examination data in sensitivity analyses; results were materially unchanged. Also pertaining to IDEFICS/I.Family: to avoid collinearity among dietary variables, each food group exposure was examined in a separate model. All primary models were adjusted for sex, age at examination, parental education, and prenatal smoking. Additional adjustment for early-life factors (birthweight and breastfeeding) was explored in sensitivity analyses, yielding similar results.
